# New Insights in the Long-Term Atmospheric Corrosion Mechanisms of Low Alloy Steel Reinforcements of Cultural Heritage Buildings

**DOI:** 10.3390/ma10060670

**Published:** 2017-06-19

**Authors:** Marie Bouchar, Philippe Dillmann, Delphine Neff

**Affiliations:** 1 Saint-Gobain Recherche, 39 quai Lucien Lefranc, 93303 Aubervilliers CEDEX, France; marie.bouchar@saint-gobain.com; 2 LAPA-IRAMAT, NIMBE, CEA, CNRS, Université Paris-Saclay, CEA Saclay, 91191 Gif-sur-Yvette, France; delphine.neff@cea.fr

**Keywords:** cultural heritage metals, iron, low alloy steel, atmospheric corrosion, in-situ measurement, micro-Raman

## Abstract

Reinforcing clamps made of low alloy steel from the Metz cathedral and corroded outdoors during 500 years were studied by OM, FESEM/EDS, and micro-Raman spectroscopy. The corrosion product layer is constituted of a dual structure. The outer layer is mainly constituted of goethite and lepidocrocite embedding exogenous elements such as Ca and P. The inner layer is mainly constituted of ferrihydrite. The behaviour of the inner layer under conditions simulating the wetting stage of the RH wet/dry atmospheric corrosion cycle was observed by in situ micro-Raman spectroscopy. The disappearance of ferrihydrite near the metal/oxide interface strongly suggests a mechanism of reductive dissolution caused by the oxidation of the metallic substrate and was observed for the first time in situ on an archaeological system.

## 1. Introduction

Since Antiquity and the Middle Ages, stone buildings have been reinforced by metallic clamps, rods, and ties. Recent studies have demonstrated that thousands of kilograms of ferrous alloys have been used since their inclusion in buildings in monuments of the Middle Ages, such as cathedrals [1,2,3,4]. Today, these reinforcements are considered as part of heritage buildings and must be preserved as testimonies of the skills of ancient builders. Most of these artefacts are exposed to atmospheric corrosion, either indoor or outdoor [5]. Besides, numerous archaeological artefacts are conserved in museum storage rooms, and in some cases, are submitted to uncontrolled environmental conditions. For that reasons, for several years, the very long term atmospheric corrosion of iron and steel has been studied, especially on these monuments. Another reason for the study of these kind of corrosion systems is that they can be considered as analogues for the prediction of the corrosion behaviours of today’s materials that are going to be used for very long periods in civil engineering or the nuclear industry [6,7]. 

Former characterisation studies performed on ferrous metals coming from historical monuments [8,9,10,11] observed that the layers developed in such conditions over several hundred years were a mix of Fe-containing phases such as goethite (α-FeOOH), lepidocrocite (γ-FeOOH), akaganeite ((β-FeO_1-x_(OH)_1+x_,Cl_x_), feroxyhyte (δ-FeOOH), ferrihydrite (Fe_2_O_3_,1.8H_2_O), maghemite (γ-Fe_2_O_3_), magnetite (Fe_3_O_4_), and hematite (Fe_2_O_3_), whose proportions depend on the initial state of the alloy (presence of a former scale due to the elaboration process) and the corrosion conditions (wet/dry cycle, presence of pollutants,…). Nevertheless, the exact corrosion mechanisms involved in these long-term systems are still under discussion. Most authors admit that it is based on the well-known RH (relative humidity) “wet/dry” cycle that occurs in the case of atmospheric corrosion, but the different processes involved during this stage are not completely deciphered. 

The wet/dry cycle is divided into three steps: (i) the wetting stage, when the electrolyte progressively covers the surface of the material (here, the corrosion product); (ii) the wet step, when a continuous layer is covering the surface; and (iii) the drying step, when the electrolyte evaporates from the surface. In the first step, it is assumed by some authors [12,13,14,15] that the Fe(III)-phases constituting the corrosion product layer (CPL) participate in the oxidation of the metal. This was suggested for short-term [16,17] and long-term corrosion systems [18,19,20,21]. These Fe(III)-phases are lepidocrocite [22,23], ferrihydrite [9,24] and, to a lesser extent, ferroxyhite and akaganeite. Some authors made the hypothesis that these phases could be reduced into conductive ones. A different hypothesis was suggested for the reduced phase that is obtained: solid state transformation into an Fe(II)-phase γ-Fe·OH·OH, with a structure similar to a hydrogel [23], dissolution, reduction, and re-precipitation into Fe(II)-containing species [20]. Monnier et al. [21] demonstrated that the nature of the final phase (a mixture of magnetite and Fe(II)-hydroxide) depends on the reduction mode (current or potential imposed) and on the electrolyte pH. Other studies [25,26] suggest that the anodic and cathodic reactions are decoupled in thick CPLs (the latter one being located at the outer part of the CPL), supposing the existence of transient conductive phases formed during the wetting stage by the reduction of electrochemically reactive phases. This transient phase is re-oxidised at the end of the cycle (see below).

Nevertheless, despite all these different studies, the reduction of reactive phases inside ancient corrosion thick layers was never observed directly. During the following steps (wet step) of the wet/dry cycle, it is admitted that dissolved O_2_ is transported in the pores of the CPL and reduces when it meets species that can be oxidised (Fe^2+^ ion dissolved after the reduction of an electrochemically reactive phase during the first stage, Fe(II) conductive phase, or even the metallic substrate). In the case that conductive species are present, the behaviour of the system would completely change, allowing a decoupling of anodic and cathodic oxygen reduction reactions. At the end of the cycle, the massive supply of oxygen and the possible drying of the pores lead to the oxidation of all Fe(II)-species present in the system. At least part of the pores of the CPL are empty of electrolyte.

Considering this short state of the art, the aim of the present study is twofold. First, a supplementary long-term corrosion system is studied: the iron clamps corroded outdoors on the Metz cathedral, since the 15th centuries. Because of the potential variability of the ancient corrosion layers, it is of primary importance to extend the set of ancient systems analysed with fine characterisation methods. One important issue is to estimate the proportion of potentially reactive phases inside the layer and their location. The other aim of the study presented in this paper is to perform an in situ experiment on the corrosion system to simulate the wetting stage and to follow the possible reduction of reactive phases by micro-Raman spectroscopy. 

## 2. Materials and Methods 

### 2.1. Set of Samples

**Figure 1:**
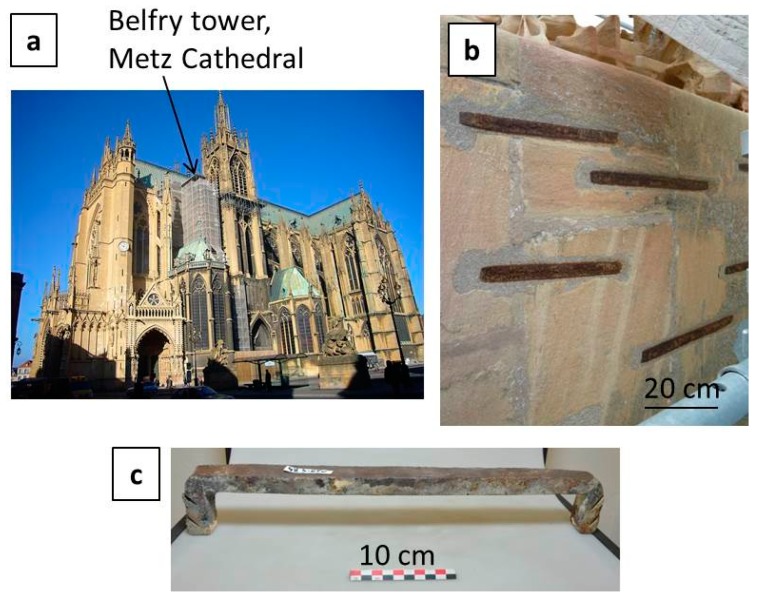
(**a**) Metz Cathedral; (**b**) iron reinforcement clamps located outdoors on walls of the La Mutte tower; (**c**) one of the iron clamps studied at the laboratory.

Samples were taken on iron clamps removed from the belfry tower of the Metz Cathedral (France) during a restoration campaign (2010–2014) (Figure 1a). These clamps were put in the monument during the building (15th centuries AD), as demonstrated by archaeological science studies [1,3]. The clamps are about 40 cm long and have a rectangular section of 3 cm by 2 cm (Figure 1c). Because of the heterogeneity of ancient iron and steel [4], the average chemical composition is a value of low significance. Investigations made by EPMA on the metallic matrix reveal that except P and C, all of the elements have contents below 100 ppms. The metal was studied by metallographic observations on cross sections (see Supplementary Materials). It is composed of low alloy steel (wt. % C < 0.3) containing several thousand ppm of phosphorus. Some non-metallic slag inclusions reaching several 100 µm in size are also observed in the metallic matrix. These clamps are located outdoors on walls, in a vertical position (Figure 1b). Meteorological data collected daily by the Metz-Frescaty weather station from 1981 to 2010 give an accurate estimate of the environmental conditions. The RH ranges between 70% and 90%, and the temperature ranges between 1 °C and 19 °C.

Complete cross sections of the clamps were performed, using a slow speed diamond saw. These samples were mounted in epoxy resin, and prepared by grinding (SiC, grade 80–4000) and polishing (diamond paste, 3 and 1 µm) under ethanol. 

### 2.2. Characterisation Methods

The entire corrosion system corresponding to the outer face of the clamp, exposed to atmospheric corrosion, was observed on transverse sections by an Optical Microscope (OM) and by a Field Emission gun Scanning Electron Microscope (FESEM) JEOL 1200. The chemical composition of the CPL was analysed by EDS (IdFix™ and Maxview™ softwares, Fondis Electronic, France) coupled to FESEM (accelerating voltage of 15 kV). 

The structure of the CPL was investigated by micro-Raman Spectroscopy (µRS) on the cross sections using a Renishaw InVia spectrometer equipped with a frequency doubled Nd:YAG laser at 532 nm. The laser was focused on the sample thanks to a Leica DM/LM microscope. With the ×50 focus used for the acquisitions, the beam diameter is 1.5 µm and the penetration length is about 2 µm. The spectral resolution given by the CCD detector is 2 cm^−1^. As some iron oxides are very sensitive to laser exposure, density filters are used to control the laser power on the sample under 100 µW. Spectral acquisitions are performed with the Renishaw Wire 3.2 software in an ultra-fast mode (StreamLine™, Renishaw, UK).

The iron oxides and oxyhydroxides constituting the rust layer were quantified on spectra extracted from maps or acquired locally on the corrosion product layer (CPL) using a program developed in our laboratory, inspired from the CorATmos program developed by Monnier et al. [9], and adapted to process data of maps with a very large number of spectra. This program, based on linear combinations of reference spectra of pure phases, fits each experimental spectrum between 200 and 800 cm^−1^ with a Levenberg-Marquardt algorithm in order to reach the proportion of the phases at each point of a map. The reference spectra are those used by Monnier et al. The error has been estimated by the latter authors as about 5% for each quantified phase. More details on the procedure can be found in [11].

### 2.3. In Situ Experimental Setup

**Figure 2:**
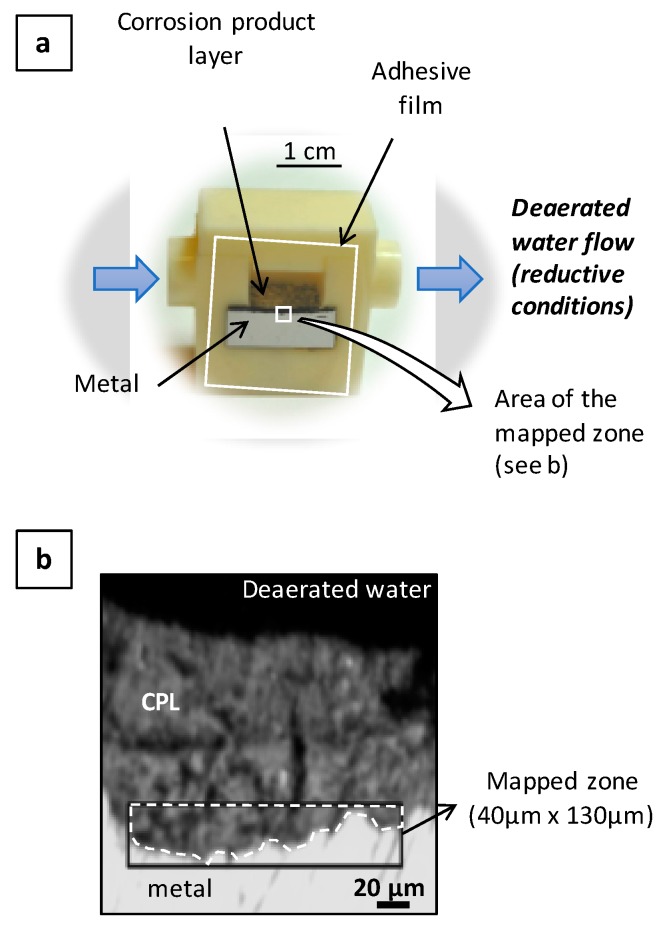
(**a**) In situ experimental cell; (**b**) Optical microphotograph of the transverse section. Black rectangle: zone observed by µRS maps. Dotted line: zone where the phase quantification is performed. CPL: Corrosion Product Layer.

The in situ cell (Figure 2) is designed to allow, at the same time, the circulation of deoxygenated water on the surface of the CPL and µRS analyses on a cross section of the CPL. A transverse section of the clamp of about 1 cm^3^ is mounted inside the cell and then polished. After polishing, an adhesive film (HD ClearTM Crystal Tape, Duck^®^ Brand, USA) transparent to visible light and a laser beam is put on the cross section, avoiding the solution to bypass the corrosion layer and to directly reach the bare metal. The Raman spectrum of the adhesive film only shows two bands (810 cm^−1^ and 398 cm^−1^) in the region of the corrosion product signals (200–850 cm^−1^), so its contribution can be easily removed from the Raman spectra obtained on the CPL.

## 3. Results

### 3.1. Characterisation of the Corrosion Products

**Figure 3:**
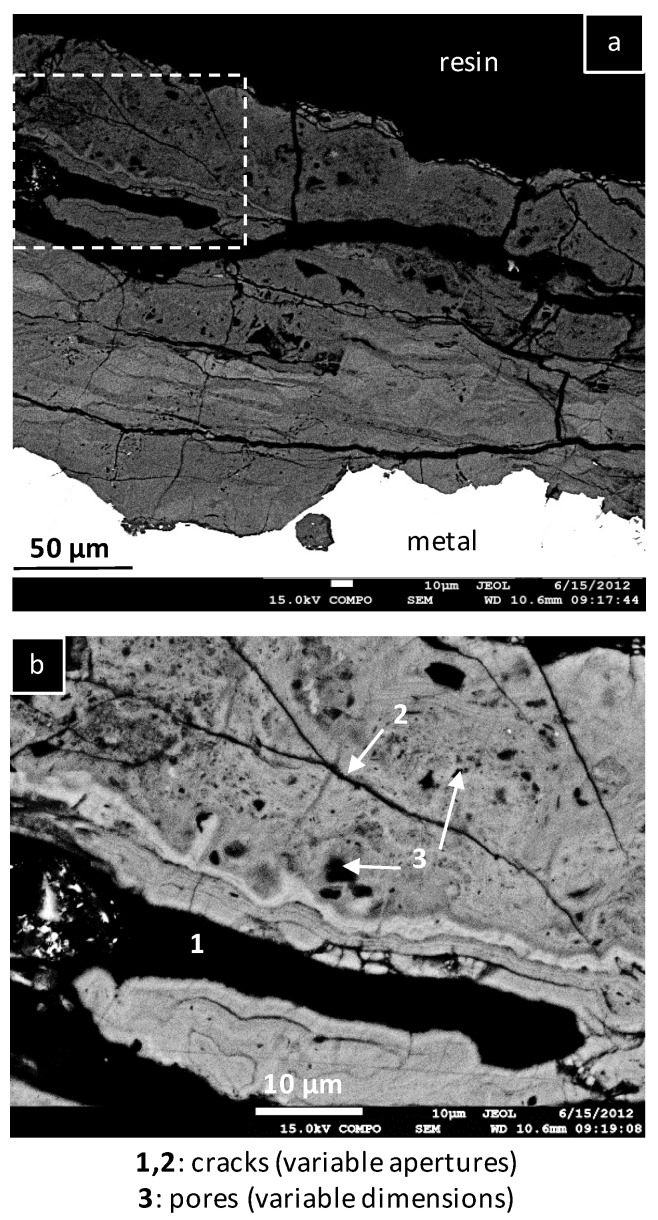
FESEM images of the corrosion product layer in the backscattered electron mode. Image (**b**) corresponds to a magnification in the white dot rectangle drawn on the image (**a**). 1&2: cracks with various apertures; 3: pores with various sizes.

The average thickness of the CPL has been estimated on a cross section from 154 measurements performed with a regular step covering a CPL length of 12 mm along the metal/CPL interface. The average value is 110 ± 50 μm. The local values are dispersed and vary between 20 and 260 µm. The CPL is crossed by many cracks with variable orientations relative to the metal/CPL interface. The crack aperture ranges from less than 1 µm to a few micrometers (Figure 3a). These cracks may have formed by the effect of mechanical strengths generated by corrosion on the metal, leading to a volume increase when iron oxides replace metallic iron. Moreover, the CPL presents a network of micrometric and submicrometric pores (Figure 3b). As stated by other authors [8,10] on ancient corrosion layers, the CPL seems to be bi-layered with an internal zone much more dense (despite presenting micro-cracks) than an outer and more porous layer.

**Figure 4:**
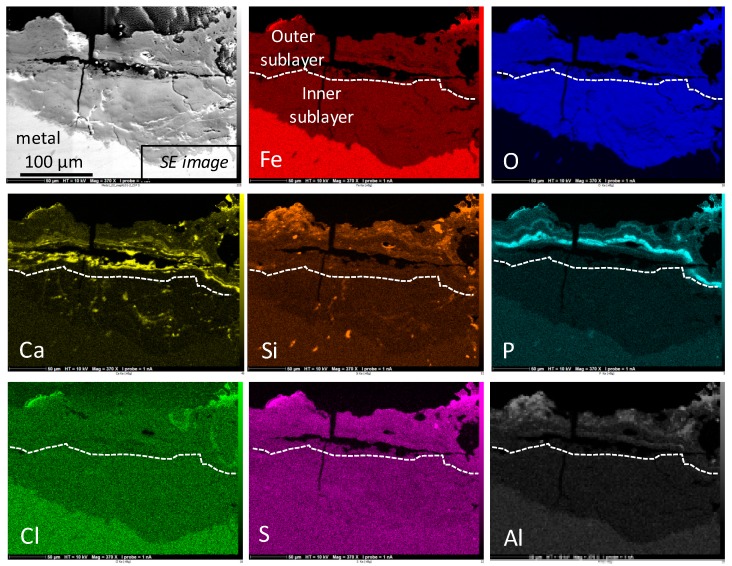
FESEM image of the corrosion product layer in the backscattered electron mode, and related EDS maps (Kα lines) of detected chemical elements. The white dot line corresponds to the limit between the inner and outer sublayers of the corrosion product layer.

The FESEM/EDS maps performed on cross section samples confirm the structuration of the CPL into two sublayers with different elemental compositions (Figure 4). In both the inner and outer sublayers, the major elements are iron and oxygen. The sublayers differ from each other by their average contents in minor elements. Their distribution is quite inhomogeneous inside each sublayer. Calcium is mainly located along the cracks and pores in both sublayers, with the contents reaching 10 wt. % locally. This minor element corresponds to an exogenous pollution, as calcium is found in high amounts in stone and mortar dusts, as well as particles from monument walls in which iron clamps were sealed. Silicon is essentially located in the most external part of the CPL. As calcium, it is assumed that silicon comes from building walls. Some spots can nevertheless be identified in the inner sublayer. They correspond to non-corroded slag inclusions initially present in metallic matrix (see [3] and “Materials and methods” section). Chlorine is only present in rare zones in the outer sublayer, with higher contents reaching 1 to 2 wt. %. Even far from the seacoast, as in the present case, low quantities of this element can be found in the atmosphere, due to anthropogenic sources, leading to its accumulation in the corrosion product. Almeida et al. [27] showed in a corrosion study on 19 urban and rural sites conducted in 2000, that even with low deposition rates of chlorides (<3 mg·m^2^·d^−1^), Cl- can be detected in the corrosion products at levels higher than 308 mg·m^2^, with most of the values being comprised between 34 and 66 mg·m^2^. Sulphur is detected only in the extreme outer sublayer (sometimes reaching about 1 wt. %). This element comes from the SO_2_ of urban and industrial atmospheres [28]. The level of this compound is controlled for several decades and the annual mean concentration is now well below 100 µg·m^−3^; however, at the end of the XIXth centuries and during the XXth centuries, this value could reach several 100 µg·m^−3^ [29]. The phosphorus content may locally reach several weight percents (2 to 10 wt. %) in the outer sublayer. The zones rich in phosphorus correspond to one or several strips parallel to the metal/CPL interface. The presence of phosphorus in the metallic substrate may not explain the high contents in the CPL alone. The most probable hypothesis to explain the origin of phosphorus in the outer sublayer is the contribution of an exogenous source. Animal excrement residues rich in phosphates could be one of these sources [30]. The phosphorus content in the inner sublayer is lower than 0.5 wt. %.

**Figure 5:**
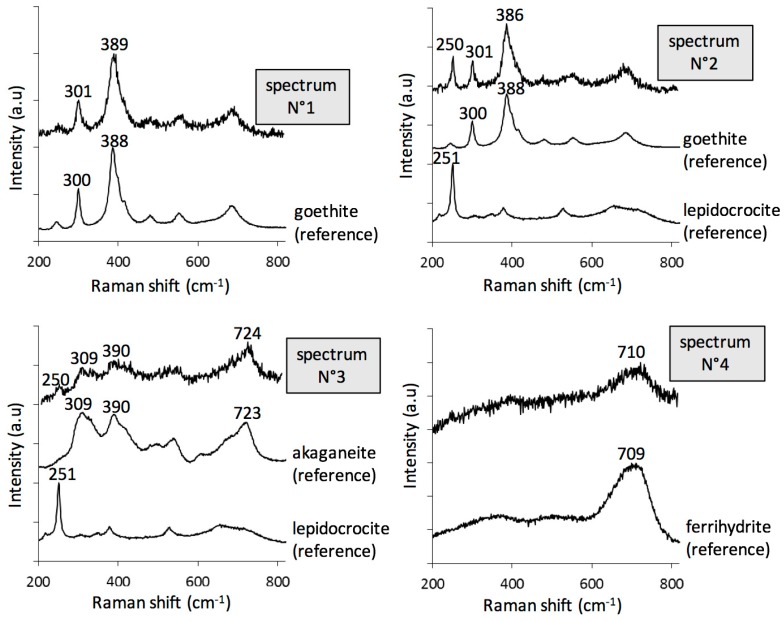
Set of Raman spectra acquired on the corrosion product layer. Each spectrum is plotted together with the corresponding ‘reference’ spectra. (spectrum N°1): goethite; (spectrum N°2): blended goethite/lepidocrocite; (spectrum N°3): blended akaganeite/lepidocrocite; (spectrum N°4): ferrihydrite.

The CPL phases have been identified on a cross section by µRS. At the scale of the laser probe, most of the spectra present bands corresponding to a mixture of several phases. Figure 5 shows a set of spectra testifying the presence of four oxyhydroxides in the CPL:

- goethite α-FeOOH (bands at 300 and 390 cm^−1^, spectra N° 1–2);
- lepidocrocite γ-FeOOH (intense peak at 250 cm^−1^, spectrum N° 2);
- akaganeite β-FeO_1-x_(OH)_1+x_,Cl_x_) (combination of asymmetric bands at 309, 390, and 724 cm^−1^, spectrum N° 3), only present in rare zones;
- ferrihydrite Fe_2_O_3_,1.8H_2_O [31], and more generally hydrated oxyhydroxides (broad and symmetric signal, without shoulder, around 710 cm^−1^, spectrum N° 4). In fact, feroxyhite (δ-FeOOH) also presents a broad band in the same region as ferrihydrite, but at 677 cm^−1^ [32]. The Raman spectra obtained on the Metz cathedral samples are mainly in agreement with ferrihydrite, but the presence of small amounts of feroxyhite cannot be completely excluded.

Maghemite (γ-Fe_2_O_3_) that presents a sharp double peak at 670 and 720 cm^−1^, is very different from the broad band observed on the experimental spectra at this location, whilst magnetite (Fe_3_O_4_) and hematite (α-Fe_2_O_3_) were not detected in the CPL. 

**Figure 6:**
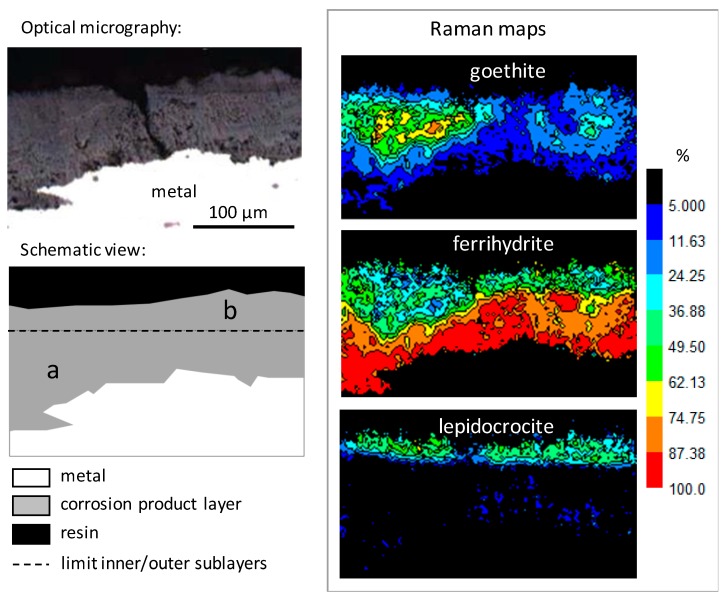
Quantitative µRS maps on a corrosion product layer containing goethite, ferrihydrite, and lepidocrocite. The dot line on the schematic view corresponds to the average limit between the inner sublayer (a) and outer sublayer (b).

µRS quantitative maps (see “Materials and methods” section) also highlight the CPL structuration into two sublayers. Figure 6 presents a map acquired in a representative zone of the CPL. The limit between the inner and outer sublayers is shown by a black dot line on the schematic view of the map. The phase quantifications in both zones delimited by this line (zones a and b) are given in Table 1. The inner sublayer contains mainly ferrihydrite—contents higher than 70%—and goethite—about 5 to 25%. Lepidocrocite is present in small amounts (generally <10%), but only in a few islets. The outer sublayer also contains goethite in similar quantities as the inner one. However, this layer is much poorer in ferrihydrite—with local contents ranging between 0 and about 50%—and much richer in lepidocrocite—with contents of about 25%, locally reaching 70%. In rare limited zones of the outer sublayer, akaganeite is detected in quantities lower than 10%. Lastly, it was not possible to detect by µRS a specific phosphorus-containing phase in the most phosphorous zones of the outer layer. This is probably due to the fact that the phosphorous products are not crystallised as stated by other authors for ancient corrosion products [33,34]. All the results are summarised in Table 2.

### 3.2. In Situ Reactivity Test of the Corrosion Layer

A specific sample, with the same corrosion pattern as the one evidenced in Section 3.1, was selected to perform a reduction test on the CPL. This test aims to show that reactive phases (here, mainly ferrihydrite) in contact with the metal are susceptible to playing the role of an oxidizer in the absence of dioxygen and thus to being reduced. The experimental setup and method used for the test are described in the “Materials and methods” section. 

**Figure 7:**
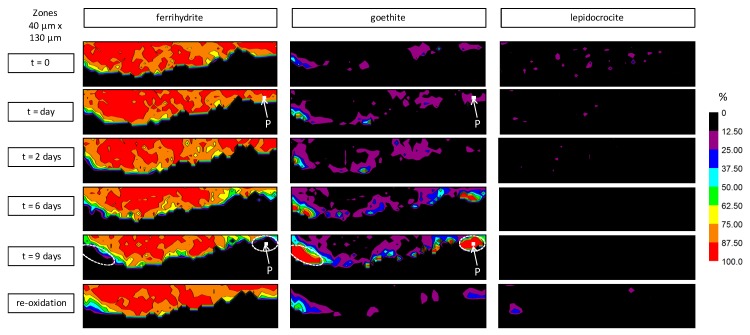
Quantitative µRS maps in the zone identified by the dot line on Figure 2b: before the test (*t* = 0), after 1, 2, 6, and 9 days of deaerated water circulation, and after re-exposure to ambient air during one day (re-oxidation). The regions circled by a dot line correspond to those where ferrihydrite has disappeared after nine days of deaerated water exposure. The spectra corresponding to point P, for one and nine days, are presented in Figure 8.

**Figure 8:**
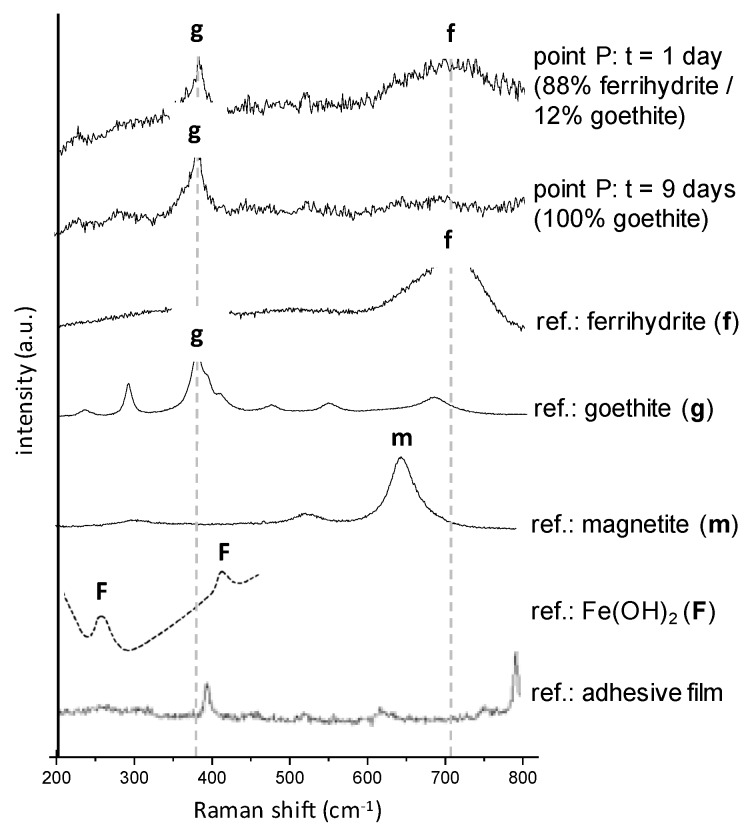
Raman spectra at point P identified on Figure 7, after one and nine days of exposure to deaerated water. The ‘reference’ spectra of the pure phases are also presented (ferrihydrite, goethite, and magnetite from [9]; Fe(OH)_2_ from [35]; adhesive film). The contribution of the adhesive film of the cell was substracted from the experimental spectra.

**Figure 9:**
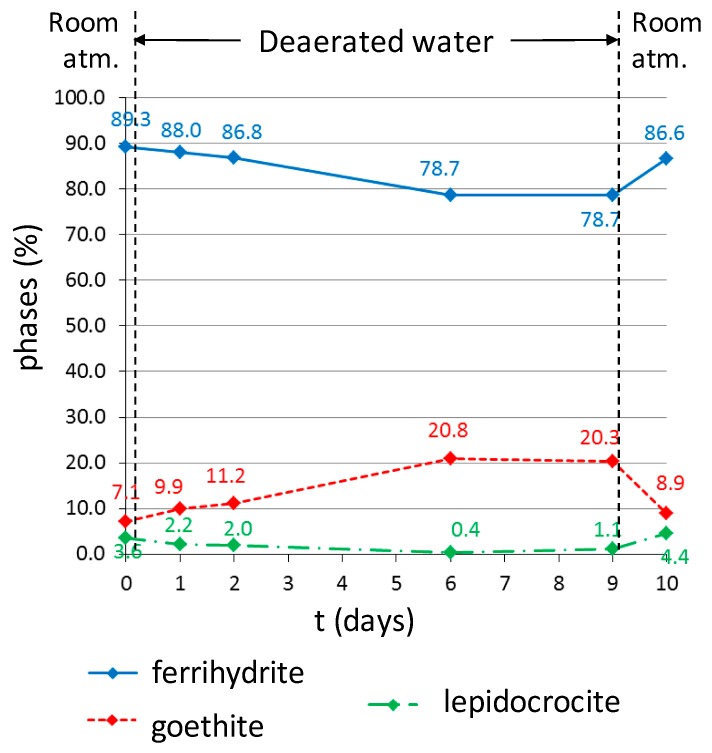
Global average contents (%) of the phases detected in the zone marked with a black rectangle on Figure 2b, calculated from the maps presented in Figure 7.

The evolution of the CPL of a clamp sample, set in a cell, has been observed by µRS at the metal/CPL interface over nine days (Figure 2). Spectral maps of the same zone have been acquired during the experiment: (i) before the introduction of deaerated water in the cell; (ii) after 1, 2, 6, and 9 days of deaerated water circulation; (iii) after stopping the flow of deaerated water, then exposure to ambient air, for one day—see Figure 7 and Figure 8. The mapped zone near the metal/CPL interface is 130 µm long and 40 µm wide—i.e., 52 × 16 points—with a step of 2.5 µm in length and width (Figure 2b). It is worth noting that this zone does not present any crack detectable by OM and FESEM, which could link directly the metal/CPL interface to the external environment. From each quantitative map acquired during the experiment, an average phase content of the entire zone can be deduced. The evolution of this average content versus time is presented in Figure 9.

At *t* = 0, before the beginning of deaerated water circulation through the experimental cell, the mapped zone mainly contains ferrihydrite (about 90% average spread over the entire zone, and thus in contact with the metal) (Figure 7 and Figure 9, *t* = 0). Goethite (about 7% on average) and lepidocrocite (about 3% on average) are minor phases and are present as localized islets. After one day of deaerated water circulation in the cell, quantitative maps do not present any significant difference with those obtained at time *t* = 0. However, for longer treatment times, the obtained µRS maps are quite different. After two days, goethite islets have expanded spatially and lepidocrocite ones have disappeared. Between two and six days, the average contents of ferrihydrite and lepidocrocite decrease progressively, whereas the average goethite content increases (Figure 9). After six days in deaerated water, overall content variations are significant compared to the maximal quantification error, which is estimated to be 5% (see “Materials and methods” section). The ferrihydrite content has decreased by more than 10%, whereas the goethite content has increased by more than 10%. Then, the CPL composition stabilizes. After nine days in deaerated water, the quantification results are similar to those obtained at time *t* = 6 days. Quantitative maps of phases at time *t* = 9 days show that variations of ferrihydrite and goethite contents are localised at some specific places in the CPL. The highest variations—up to 90%—are mainly concentrated in two regions, both in contact with the metal/CPL interface. In both regions, ferrihydrite has disappeared and the goethite relative content has increased. Figure 8 compares the spectra acquired at times *t* = 1 day and *t* = 9 days at the same point in one of these two regions (labelled “P” on Figure 7). The broad band around 700 cm^−1^, corresponding to the presence of ferrihydrite and visible on the spectrum at *t* = 1 day, is no longer detectable on the spectrum at *t* = 9 days.

No other new phases than the ones present at time *t* = 0 have been detected during the test. Specifically, magnetite and iron hydroxide (II), detected by Monnier et al. by X-ray absorption spectroscopy and X-ray diffraction during ferrihydrite reduction induced by an electric current or potential [21], are not identified in this test on spectra from the map at *t* = 9 days. The decrease in the local ferrihydrite content is only balanced by an increase in the local goethite content. 

## 4. Discussion

**Figure 10:**
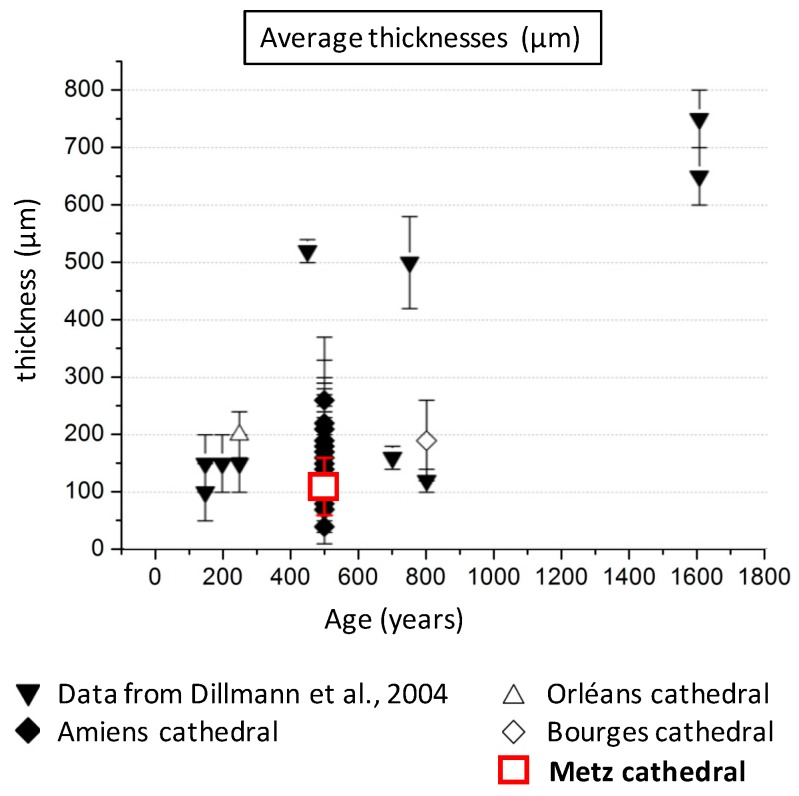
Average thicknesses and standard deviations of CPL measured on ancient low alloy steels in monuments v time. Black down triangle: from [8], losanges: Amiens cathedral [10], up open triangle: Orléans Cathedral [36], open losange: Bourges cathedral [11], red square: Metz cathedral, this study.

The chemical and structural features of the CPL from the Metz cathedral are relatively similar to the one measured by precedent studies on a reinforcement made of ferrous alloy from monuments of several hundred years old. Figure 10 compares the thicknesses measured on the samples of the Metz cathedral with the ones found on low alloy steel reinforcements of cultural heritage monuments with different ages. Values found in the present studies correspond to the lower ones measured on monuments of the same age (Amiens cathedral). An important difference to note here is that contrary to all of the other studied monuments, the clamps of the Metz cathedral were not corroded indoors. Consequently, these relatively low values could partly be due to the fact that a non negligible part of the outer zone of the layer may have been eliminated by leaching processes under the falling rain.

Nevertheless, despite this possible leaching, the outer layer is at least partly preserved, as demonstrated by the presence of the typical bi-layered structure evidenced on the cross sections. This morphology has already been observed by several authors on low alloy steels [8,11] and weathering steels [37,38] corroded during decades and centuries under comparable conditions. The outer layer contains typical exogenous elements coming from the atmosphere (S, Cl) or environment (Ca, Si, …). Some hypotheses about the formation of the bi-layered structure have been made by Burger et al. [39]. These authors examined low alloy steel coupons preliminary covered with a thin gold layer in order to mark the original surface and corroded in a climatic chamber during several months. After the corrosion test, the gold layer was located exactly between the two sublayers; the inner one mainly constituted of ferrihydrite, the outer one of lepidocrocite, as for the Metz samples. The authors deduced from these observations that the growth of the corrosion layers happened not only in the detriment of the metallic substrate (forming the inner layer), but was also governed by the dissolution of some iron cations and subsequent precipitation at the external interface of the rust layer. This dissolution/precipitation mechanism would explain the porous nature of the outer layer and the fact that important quantities of exogenous elements are entrapped in it. 

Concerning the constitutive phases, the CPL of the Metz cathedral contain the compounds usually reported by characterisation studies on such systems: goethite (α-FeOOH), lepidocrocite (γ-FeOOH), akaganeite (β-FeO_1-x_(OH)_1+x_,Cl_x_), and ferrihydrite (Fe_2_O_3_,1.8H_2_O) were identified. Nevertheless, some differences must be noted. The CPL of the Metz cathedral clamps contain significantly less akaganeite (1%) than those of the reinforcements coming from other monuments, such as the Amiens cathedral (20% for some samples) [10]. However, Metz is very far from the seacoast (350 km as crow flies) compared to Amiens (only 60 km as crow flies). The marine atmosphere must favour the formation of akaganeite in the case of Amiens, but has no influence in the Metz case. The presence of chlorine-containing phases in the CPL of Metz should more probably be due to anthropogenic polluting compounds like de-icing salts or chlorofluorocarbons (CFCs) released in the atmosphere by industries [40]. 

Another difference concerning the structure of the Metz corrosion pattern compared to former studies is the high content of ferrihydrite detected in the inner sublayer (up to 80% of the corrosion products for some samples). As a comparison, the ferrihydrite (and feroxyhite) contents in the CPL of Amiens and Bourges samples reached up to 65% and 43%, respectively. The identification of ferrihydrite on the Raman spectra is sometimes not obvious in the case of mixed phases. Specifically, its distinction with feroxyhyte, which also presents a broad band at 677 cm^−1^, may be hard [32]. However, the typical band observed on the spectra around 700 cm^−1^ in the case of the Metz samples CPL tends to favour the hypothesis of the presence of ferrihydrite instead of the one of feroxyhyte. Despite the fact that the presence of feroxyhyte cannot be completely excluded, it seems to be the minority and, for simplification reasons, only ferrihydrite will be considered in the following. This choice is reinforced by the fact that Monnier et al. also preferentially identified by XAS studies ferrihydrite rather than feroxyhyte in corrosion products formed on phosphoric iron [41].

The presence of amorphous or low-crystallised phases (such as ferrihydrite) is often reported in publications concerning the long-term atmospheric corrosion of iron and weathering steel [10,11,42]. Nevertheless, the present observation of massive quantities of ferrihydrite on 500 year-old samples is not in agreement with the hypothesis of several authors [39,43], who proposed that ferrihydrite that is one of the major phases in the first stage of the atmospheric corrosion process, progressively transforms into more crystallised phases as goethite because of reduction/dissolution/precipitation mechanisms (see below) during the wet/dry cycles. This is clearly not the case here. This confirms that the age of the sample is not the predominant parameter that influences the proportion of phases in the presence of long-term atmospheric CPL. A hypothesis to explain the major presence of this phase after several hundred years could be the fact that it contains certain amounts of phosphorus, an element that is well-known to reduce the reactivity of the corrosion products [31,44,45,46,47,48]. This point is detailed below.

Contrary to former studies on long-term atmospheric corrosion, ferrihydrite is not present in the CPL under the form of marblings surrounded by goethite (as for example in samples from the Amiens and Bourges cathedrals). Here, it is present under the form of a homogeneous layer mixed at a microscopic scale with other minor phases. As said before, several thousand ppm of P were locally detected in the inner layer. Some authors have proposed that this element could be adsorbed at the surface of ferrihydrite [33], lowering the electrochemical reactivity. Nevertheless, ferrihydrite is reported in the literature as one of the most electrochemically reactive substances during the wet/dry cycle of atmospheric corrosion [18]. As explained in the introduction part, oxygen does not reach the metal/oxide interface during the wetting stage of the RH cycle. During this stage, iron is susceptible to oxydize by reducing ferrihydrite, provided that this reactive phase is electrically connected to the metallic substrate [21]. In this case, the reduction equation would be the following:

Fe_2_O_3_,1.8H_2_O_(CPL)_ + H^+^ + e^−^ ⇔ Fe(OH)_2_ + 1.8H_2_O (1)

The disappearance of ferrihydrite during the in-situ test can be explained by its transformation either into a non-detected phase or into a soluble phase. Then, the relative contribution of the remaining phase (goethite) to the Raman spectra would increase. Ferrihydrite has a very low solubility (K_s_ = 10^−39^ at 25 °C) [48,49]. Consequently, the quantity of dissolved Fe^3+^ ions should be very low (<10^−6^ mol/L for 2 < pH < 12). In reducing media, such as in the present case, the solubility may increase by several orders of magnitude [50]. This is mainly due to the reduction of the structural Fe(III)-species into Fe(II)-species which are much more soluble than the Fe(III) ones. The reductive dissolution of ferrihydrite could thus be envisaged. In that case, the fact that the disappearance of this phase is observed by µRS, mainly in zones neighbouring the metal, strongly suggests that the main reduction process is due to the oxidation of the metal and not to the one of Fe species dissolved in water. In that latter case, the disappearance of the ferrihydrite should have been detected in the entire layer and not only at the metal/CPL interface. It has been noted by several authors that ferrihydrite especially shows a high elechtrochemical reactivity [21,51]. It was observed that at pH = 7.5 and under a potential of −1 V/SCE, a synthetic ferrihydrite is reduced into a mix of 80% magnetite (α-Fe_2_O_3_) and 20% Fe(OH)_2_ [21]. At the same pH but under an imposed current of −100 µA/mg, the same phase was mainly reduced under the form of Fe(II)-hydroxide (Fe(OH)_2_). This latter phase, less crystallised, was identified only by X-Ray absorption techniques under Synchrotron Radiation. With the µRS used in the present study, Fe(OH)_2_ is hardly detectable. Its spectra present large and weak Raman bands. Thus, it must be present in very high quantities to be detected in a mix of phases, which seems not to be the case here. On the contrary, magnetite produces intense and very characteristic Raman bands. If present, even in low quantities, this phase should have been detected. Lastly, the hypothesis of the direct re-precipitation of goethite from species formed by ferrihydrite dissolution is not likely because of the reducing conditions. 

## 5. Conclusions

A corrosion pattern formed under the outdoor atmospheric corrosion of reinforcing clamp on mediaeval monuments for several hundred years is reported. The bi-layered corrosion product layer contains exogenous minor elements (Cl, Ca, Si, S, and P) in the outer layer and about 0.5% of endogenous P in its inner layer. The latter is mainly constituted of Ferrihydrite (77%) and Goethite (20%). The outer layer, in addition to these two phases (respectively, 44 and 21%), contains higher quantities of lepidocrocite (35%). Compared to other corrosion patterns of long-term atmospheric corrosion, the quantity of ferrihydrite is particularly high.

A simulation of the wetting stage of the wet/dry cycles of atmospheric corrosion coupled to in situ µRaman observation allowed us to observe the disappearance of ferrihydrite, particularly at the metal/CPL interface. No neo-precipitated compounds could be evidenced. The disappearance of ferrihydrite along the metal/CPL interface is caused by a reduction of this phase (before or after dissolution), either under the form of dissolved Fe^2+^ ions or under the form of a phase which is non detectable by µRS. In the first case, the dissolved Fe^2+^ ions may migrate in the pore water and be re-oxidised by the supply of solvated dioxygen during the following stages of the wet/dry cycle. In the case of the reduction of ferrihydrite under the form of a solid phase, as proposed in the literature, the “reduced ferrihydrite” could be partially conductive [18,23]. Provided that ferrihydrite is significantly reduced, the conductivity of the reduced species could lead to a decoupling of the anodic (Fe(0) oxidation) and cathodic corrosion (dissolved dioxygen reduction) reactions in the next step of the wet/dry cycle. 

The fact that the disappearance of ferrihydrite is only detected after one day suggests that the process is quite slow. This brings us to raise the question of the influence of the duration and the nature of the wet/dry cycle on this process. In the case of a short wetting stage compared to the characteristic reduction time, the quantity of reduced ferrihydrite could be negligible and consequently have only a small impact on the mechanism. Nevertheless, one should keep in mind that µRS—especially in the conditions used here (through a polymer film in an in situ cell)—could not be sensitive to small variations of phase contents. Thus, it is difficult to evaluate the intensity of the reduction process and its predominance in the case of short wetting stages. No further evolution of the system is observed after nine days of the experiment. It seems that the electrical connection with the metallic substrate is not preserved after a certain time, forbidding the reduction of Fe(III)-phases farther in the CPL. This strongly questions the hypothesis of the formation of a reduced conductive phase, allowing the decoupling of anodic and cathodic reactions. This observation more probably suggests the reductive dissolution of ferrihydrite under the form of Fe^2+^ ions. With the supply of solvated dioxygen at the end of the in situ experiment, the ferrihydrite content at the interface increases again, probably corresponding to the precipitation of this phase after the re-oxidation of Fe^2+^ ions dissolved in the pore solution. It is interesting to note that this new Fe(III)-phase is mainly ferrihydrite and not goethite.

This in-situ approach, by µRaman analyses, allowed us for the first time to observe the behaviour of a thick corrosion layer during the first stages of atmospheric corrosion. It would be very interesting in further studies to confront these observations with electrochemical approaches that would allow one to assess quantitative aspects of the reduction process and to refine the mechanisms. 

## Figures and Tables

**Table 1:** Average phase contents in zone a (inner sublayer) and zone b (outer sublayer) of Figure 6. n.d.: not detected.

	Average %			
	Ferrihydrite	Goethite	Lepidocrocite	Akaganeite
Inner sublayer (zone a)	77	20	3	<1
Outer sublayer (zone b)	44	21	35	n.d.

**Table 2:** Summing up of the characterization results.

Layer Part	Morphology	Minor Elements	Phases %
Inner	Cracks (micro and sub-micro) Multiphased Relatively dense	Ca (exogenous) along cracks (up to 10%) Si (endogenous) from former slag inclusions P (endogenous) from the metal (about 0.5%)	Ferrihydrite: 77 Goethite: 20 Lepidocrocite: 3
Outer	Cracks (micro and sub-micro) Porous	Ca (exogenous) along cracks (up to 10%) Si (exogenous) Cl (exogenous) in rare zones S (exogenous) along outer surface P (exogenous) up to 10%	Ferrihydrite: 44 Goethite: 21 Lepidocrocite: 35

## Supplementary Materials

The following are available online at www.mdpi.com/1996-1944/10/6/670/s1, Figure S1: metallographic study of the clamp.
