# Supplementary material for: New Insights in the Long-Term Atmospheric Corrosion Mechanisms of Low Alloy Steel Reinforcements of Cultural Heritage Buildings
Source: Materials (Basel). 2017 Jun 19;10(6):670. doi: 10.3390/ma10060670 (PMC5554051; doi:10.3390/ma10060670)
Supplement: Supplementary file 1 [file materials-10-00670-s001.pdf]

S1 metallographic study of the clamp

The metallographic investigations performed on the transverse section of the clamp after Nital etching reveal that the microstructure is heterogeneous but mainly composed of pure ferrite (%C < 0.02).

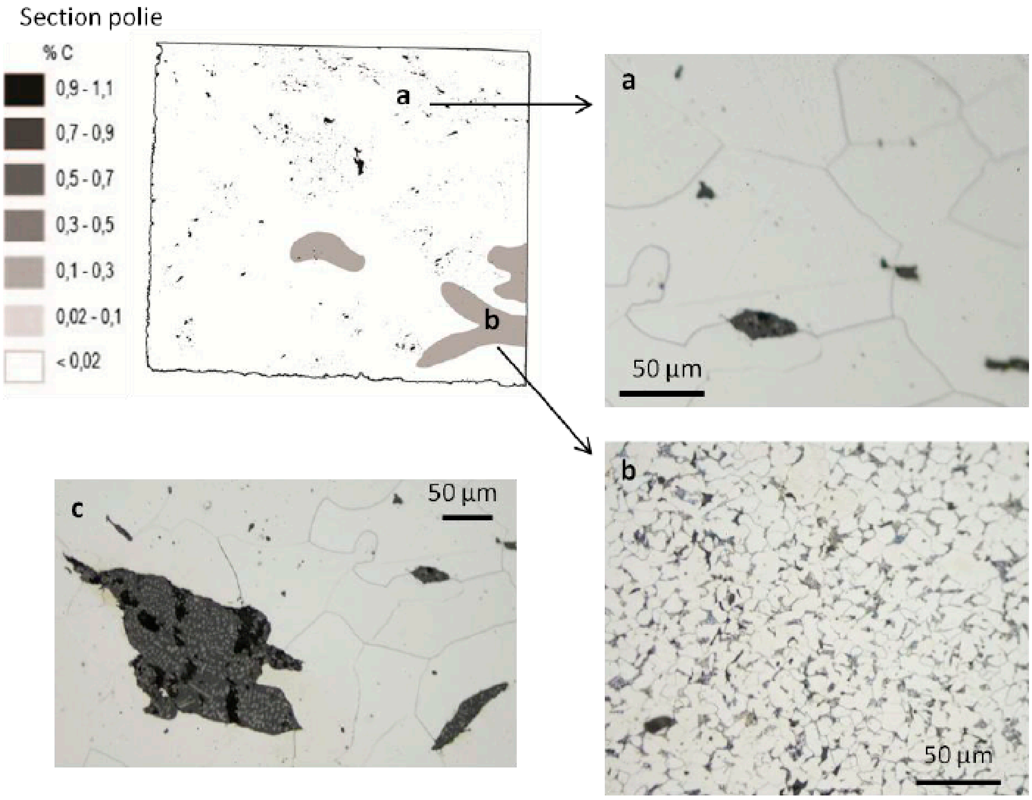

Figure 1 : transverse section of one of the analysed clamps with distribution of carbon content and associated microphotographs after nital etching.

After re-polishing and Oberhoffer etching, the presence of phosphorus in the ferritic matrix was evidenced. The observation of typical ghost-structures reveals that this element content reach several thousand ppms.

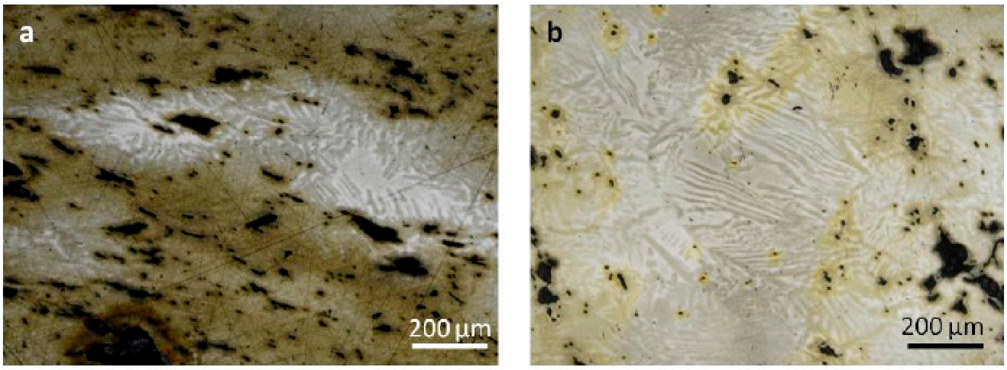

figure 62 : Micrographies optiques de 2 zones après attaque Oberhoffer sur une section polie montrant les structures fantômes (en blanc et gris) dues à la présence de phosphore dans le métal. Les micrographies a et b présentent des zones plus ou moins hétérogènes.
